# EGFR as a prognostic biomarker and therapeutic target in ovarian cancer: evaluation of patient cohort and literature review

**DOI:** 10.18632/genesandcancer.142

**Published:** 2017-05

**Authors:** Christine Mehner, Ann L. Oberg, Krista M. Goergen, Kimberly R. Kalli, Matthew J. Maurer, Aziza Nassar, Ellen L. Goode, Gary L. Keeney, Aminah Jatoi, Derek C. Radisky, Evette S. Radisky

**Affiliations:** ^1^ Department of Cancer Biology, Mayo Clinic, Jacksonville, FL, USA; ^2^ Department of Health Sciences Research, Division of Biomedical Statistics and Informatics, Mayo Clinic, Rochester, MN, USA; ^3^ Department of Medical Oncology, Mayo Clinic, Rochester, MN, USA; ^4^ Department of Laboratory Medicine and Pathology, Mayo Clinic, Jacksonville, FL, USA; ^5^ Department of Health Sciences Research, Division of Epidemiology, Mayo Clinic, Rochester, MN, USA; ^6^ Department of Laboratory Medicine and Pathology, Division of Anatomic Pathology, Mayo Clinic, Rochester, MN, USA

**Keywords:** epidermal growth factor receptor, EGFR, ovarian cancer, tissue microarray, prognostic biomarker

## Abstract

**Background:**

Limited effectiveness of therapeutic agents targeting epidermal growth factor receptor (EGFR) in clinical trials using unselected ovarian cancer patients has prompted efforts to more effectively stratify patients who might best benefit from these therapies. A series of studies that have evaluated immunohistochemical (IHC) staining of EGFR in ovarian cancer biopsies has produced unclear results as to the utility of this measure as a prognostic biomarker. Here, we used one of the largest, single institution cohorts to date to determine possible associations of EGFR expression with patient outcome.

**Methods:**

We performed IHC staining of EGFR in tissue microarrays including nearly 500 patient tumor samples. Staining was classified by subcellular localization (membranous, cytoplasmic) or by automated image analysis algorithms. We also performed a literature review to place these results in the context of previous studies.

**Results:**

No significant associations were found between EGFR subcellular localization or expression and histology, stage, grade, or outcome. These results were broadly consistent with the consensus of the reviewed literature.

**Conclusions:**

These results suggest that IHC staining for EGFR may not be a useful prognostic biomarker for ovarian cancer patients. Future studies should pursue other staining methods or analysis in combination with other pathway mediators.

## INTRODUCTION

Ovarian cancer, with only a 45% 5-year survival rate, remains one of the most devastating malignancies for women [1]. Most tumors are diagnosed at advanced stages; thus, there remains a necessity for new therapeutic targets that are effective in the context of progressive disease, as well as identification of markers that would improve clinical management of affected women.

Epidermal growth factor receptor (EGFR) is a key signaling molecule that drives cellular proliferation, migration, and invasion [2]. Selective EGFR inhibitors have been recommended as first-line therapy in lung cancer patients harboring EGFR mutations [3-5], and have also shown modest effectiveness against tumors of the pancreas [6, 7] Identification that EGFR is expressed in up to 90% of certain histotypes of ovarian tumors led to investigation of this molecule as a potential prognostic biomarker as well as therapeutic target in ovarian cancer [8, 9]. Unfortunately, response to EGFR-targeted tyrosine kinase-based inhibitors (TKIs) in unselected ovarian cancer patient populations has not been encouraging, with 0-6% response rates in patients with persistent or recurrent disease [10-12], and no significant survival benefit as a maintenance therapy for patients with response or stable disease after first-line chemotherapy [13].

The high EGFR expression found in ovarian tumors [8] and known ability of this pathway to drive tumor cell proliferation and dissemination remain compelling reasons to continue to pursue EGFR inhibitors for ovarian cancer therapy, yet the poor results seen in clinical trials to date point to a need for better methods for patient selection and stratification. Relevant criteria that may be useful in identifying responders may include histological features or molecular subtypes, disease stage, chemoresistance, as well as evidence for the expression and activation of EGFR itself.

Here we have assessed EGFR expression levels in patient-derived tissue microarrays using one of the largest, single institution ovarian cancer patient cohorts to date. We have evaluated protein staining intensity and localization, and have assessed potential significant associations with tumor stage, survival, and histology. We present our results in the context of the current literature focusing on EGFR as a biomarker in ovarian cancer. By evaluating differences and similarities in relation to our own findings, we critically discuss the suitability of EGFR staining as a biomarker and consider possible alternatives that may be more promising as prognostic biomarkers and as potential predictive markers to stratify patients for EGFR inhibitor treatment.

## RESULTS

### Clinical characteristics

From 570 patient samples, after excluding patient samples with missing data, undetermined histology, or missing tumor tissue, tissue samples representing 488 patients were included in the analysis (Table 1). The age range at diagnosis was between 21 and 93 years. Histological distribution of the tumors was similar to that reported for other cohorts [14] (high-grade serous 72.3%, endometrioid 13.7%, clear cell 6.4%, mucinous 3.3%, low grade serous 0.6%, mixed histology 3.7%). A substantial proportion (85%) of the patients presented with grade 3 disease. At a median follow-up of 116 months (range: 1-187), 339 patients (69.5%) had died. The median overall survival for the cohort was 57.8 months (95% CI: 48.4-67.5).

**Table 1:** Patient Characteristics

	Total (N = 488)
**Age at Diagnosis, years**	
N	488
Mean (SD)	61.4 (12.5)
Median	61.0
Q1, Q3	52.0, 71.0
Range	(21.0-93.0)
**Histology**	
High Grade Serous	353 (72.3%)
Low Grade Serous	3 (0.6%)
Mucinous	16 (3.3%)
Endometrioid	67 (13.7%)
Clear Cell	31 (6.4%)
Mixed	18 (3.7%)
**Stage**	
1	79 (16.2%)
2	33 (6.8%)
3	299 (61.3%)
4	77 (15.8%)
**Grade**	
1	31 (6.4%)
2	42 (8.6%)
3	415 (85.0%)
**Debulking Status**	
Missing	2
Optimal; no macroscopic disease	223 (45.9%)
Optimal; macroscopic disease <1 cm	140 (28.8%)
Optimal; macroscopic disease cm unknown	69 (14.2%)
Sub-optimal; macroscopic disease ≥1 cm	51 (10.5%)
Unknown	3 (0.6%)

### EGFR localization to membrane or cytoplasm is not associated with ovarian cancer stage, grade, or overall survival

**Figure 1:**
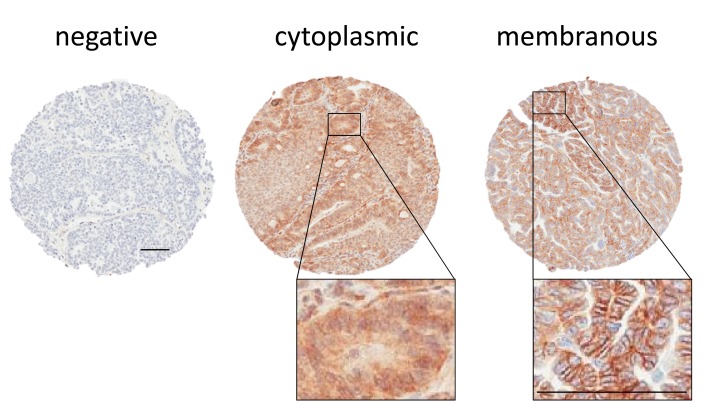
EGFR staining scored by localization in patient samples Representative stains for negative, cytoplasmic, and membranous stain are shown. All scale bars 100 µm.

We analyzed EGFR staining in our patient cohort via multiple approaches. First, we identified staining differences based on localization, as has been reported previously [9, 15-17]. We scored tissue spots as negative, membranous, or cytoplasmic (Figure 1). We found 254 patients with membranous stain, 174 patients with cytoplasmic stain and 60 without staining. Membranous expression of EGFR has been linked to elevated proliferation as well as higher stage and grade in some other studies [9, 18-21], but in our cohort, we found no significant correlation with tumor stage or grade when comparing membranous stain to cytoplasmic and unstained patient tissue specimens (Table 2). We also assessed the relationship between membranous EGFR staining localization and patient survival, but did not find a significant difference for overall survival. We further found no significant difference for overall survival or an elevation in hazard ratio when using the Cox proportional hazard model and adjusting for stage and debulking status, which have previously been established as the major clinical predictors of outcome for this cohort [22] (Table 3). Comparing unstained *versus* stained cores (grouping cytoplasmic and membranous staining together) also failed to produce a statistically significant association with tumor stage or grade, association with survival, or elevated hazard ratio using the same statistical methods (not shown).

**Table 2:** Analysis based on EGFR staining localization

	membranous	Non-membranous/negative
**Staining**		
EGFR	254	234
Histology		
High Grade Serous	186	167
Nonserous	68	67
		*p* = 0.720
**Stage**		
Early (1&2)	59	53
Advanced (3&4)	195	181
		*p* = 0.965
**Grade**		
Low (1)	17	14
High (2&3)	237	220
		*p* = 0.892

**Table 3:** Cox proportional hazard model for membranous staining

Cox Hazard Ratio (OS)	Membranous stain	
Unadjusted	HR (CI 95%)	p-value
EGFR: Membranous Stain	0.95 (0.77-1.18)	0.627
Adjusted for Stage and Debulking		
EGFR: Membranous Stain	0.94 (0.76-1.17)	0.573
Stage: Advanced (3&4)	3.31 (2.39-4.59)	7.30e-13
Debulking: Optimal	2.04 (1.48-2.80)	1.11e-5

### Dichotomized low *versus* high EGFR expression is not prognostic for survival

**Figure 2:**
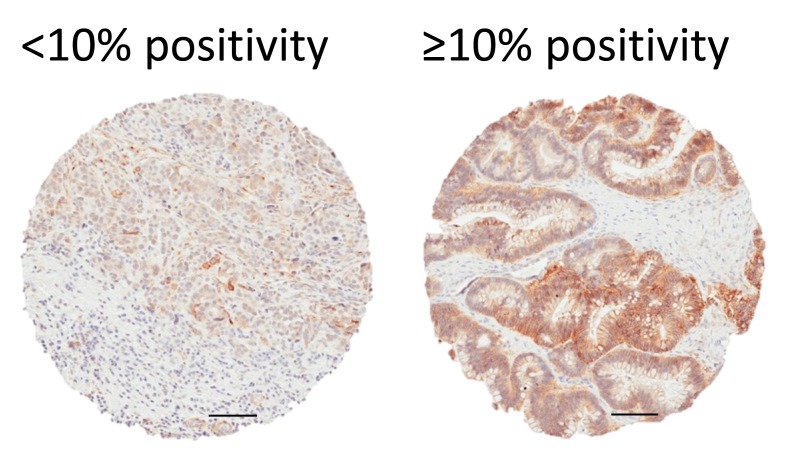
EGFR staining scored by image analysis Scores were calculated by an automated image analysis algorithm based on staining intensity and % positivity. Staining was then dichotomized into two categories: low (<10%) and high (≥10%). Representative examples for low and high staining are shown. All scale bars 100 µm.

In a separate analysis we assessed EGFR expression based on stain intensity using an automated image analysis algorithm. Calculated percent positivity scores reflect the percent of pixels exceeding a staining intensity threshold in the algorithm. Following established protocols [19, 21], we dichotomized the samples into <10% positivity (n=199) and ≥10% positivity (n=289) (Figure 2), but found no significant association of dichotomized EGFR staining with tumor histology (high-grade serous *versus* nonserous), stage, grade (Table 4), or patient survival (Table 5). We also found that positive staining for EGFR had no significant correlation with any of the other histotypes, including endometrioid, clear cell, mucinous, or low grade serous (data not shown).

**Table 4:** Analysis based on EGFR staining positivity

	Positivity	
	<10%	≥10%
**Staining**		
EGFR	199	289
**Histology**		
High Grade Serous	142	211
Nonserous	57	78
		*p* = 0.765
**Stage**		
Early (1&2)	45	67
Advanced (3&4)	154	222
		*p* = 0.970
**Grade**		
Low (1)	8	23
High (2&3)	191	266
		*p* = 0.118

**Table 5:** Cox proportional hazard model for staining ≥10% positivity

Cox Hazard Ratio (OS)	Positivity ≥10%	
**Unadjusted**	HR (CI 95%)	p-value
EGFR: ≥10%	0.94 (0.76-1.17)	0.576
**Adjusted for Stage and Debulking**		
EGFR: ≥10%	0.95 (0.76-1.19)	0.655
Stage: Advanced (3&4)	3.32 (2.40-4.61)	6.40e-13
Debulking: Optimal	2.02 (1.47-2.78)	1.66e-5

### Literature review

To place these null results in the context of prior studies examining EGFR protein expression as a prognostic biomarker in ovarian cancer we have reviewed the current literature (summarized in Table 6). Overall we find little consensus in the approaches to validate EGFR expression as a prognostic marker. Reported positive staining in ovarian tumor tissue varied widely, ranging from 9% [23, 24] to 88% [9]; contributing reasons could be differences in antibodies, tissue processing, staining techniques, and patient populations, as well as methodological differences in scoring of the EGFR tissue staining. While common histology scoring systems have been applied by some research groups, accounting for intensity and positivity, the specific details of these approaches have varied, which could substantially impact the overall analysis. Similar to our approach, some studies dichotomized staining into two groups, below and above 10% positivity [16, 19, 21, 25, 26], while others chose the more classic 2+ and 3+ score calculated from intensity and positivity [15, 18, 27-30] or have defined >1% stained cells as positive [20, 31, 32]. Ultimately, all of these thresholds are somewhat arbitrary as there has been no defined biological rationale proposed to justify which staining levels would indicate a significantly different tumor phenotype.

**Table 6:** Current literature including ovarian cancer patient tissue and EGFR staining (2000-2016)

Source [citation]	N	Country of origin	Antibody	Scoring method	Summary of results associated with EGFR expression
Alshenawy, H.A. 2010 [29]	120	Egypt	Monoclonal Clone 2-18C9 Pharm Dx (Dako) IHC	Percent positivity and Intensity	Significantly poorer OS
Brustmann, H. 2008 [30]	50	Austria	Monoclonal NCL-EGFR-384 Novocastra 1:150 IHC	Percent positivity and Intensity, Membrane localization, Large tissue sections, Serous tumors only	Significantly poorer OS
Castellvi, J. 2006 [23]	75	Spain	Monoclonal DakoCytomation 1:100 IHC	Positivity Membrane localization	No significant association with OS
Davies, S. 2014 [18]	202	USA, New Mexico	Monoclonal clone 3C6, IHC	Percent positivity and Intensity Membrane localization	No significant association with PFS
Despierre, E. 2015 [41]	218	Multicenter Europe	Cell signaling 1:50	Percent positivity and Intensity Membrane localization	No significant association with OS
Demir, L 2014 [31]	82	Turkey	Monoclonal, clone EP38Y Abcam IHC	Percent positivity Membrane localization	No significant association with OS
Elie, C. 2004 [26]	93	France	Monoclonal clone EGFR.113 Tebu 1:10 IHC	Positivity Membrane localization FIGO III or IV	No significant association with OS
Engelstaedter, V. 2012 [34]	217	Germany	Monoclonal, clone 3C6 Ventana IHC	Positivity Membrane localization FIGO III	No significant association with OS
Fujiwara, S. 2012 [28]	162	Japan	Pharm Dx (Dako) IHC	Percent positivity and Intensity	No significant association with OS Significantly poorer PFS if co-expressed with GRP30 Significantly higher expression in tumor versus borderline malignancy (n=10)
de Graeff, P. 2008 [19]	232	Netherlands	no source IHC	Percent positivity Membrane localization	No significant association with OS Significant positivity in non-serous tumors
Lassus, H. 2006 [35]	379	Finland	Monoclonal NCL-EGFR Novocastra 1:150 IHC	Positivity Membrane localization Serous tumors only	Significantly poorer OS and DFS
Lee, C.H. 2005 [24]	103	Canada	Monoclonal Clone 2-18C9 Dako IHC	Percent positivity FIGO III or IV	No significant association with DFS
Lin, C. 2009 [15]	185	Taiwan	Monoclonal, clone E30 Dako 1:25 IHC	Percent positivity and Intensity	Significantly higher expression in serous, endometrioid, clear cell, and mucinous tumors than normal tissue
Nielsen, J.S 2004 [44]	783	Denmark	Monoclonal, clone 113 Novocastra 1:40 IHC	Percent positivity Large tissue sections	No significant association with OS in univariate, significantly poorer OS in multivariate analysis, adjusting for age, FIGO stage, grade, subtype
Noske, A. 2011 [32]	121	Germany	Monoclonal Clone 5b7 Ventana Medical Systems IHC	Percent positivity Membrane localization	Significantly poorer OS for membrane stain and serous carcinoma
Psyrri, A. 2005 [16]	150	Greece	Monoclonal, clone H11 DAKO 1:50 IF	Percent positivity and Intensity Nuclear localization FIGO III or IV	Significantly poorer OS and DFS in univariate and multivariate analysis, adjusting for FIGO stage, grade, residual disease and chemotherapy response
Raspollini, M.R. 2005 [36]	60	Italy	Monoclonal Clone 31G7 Ventana Medical Systems	Positivity Membrane localization Large tissue sections FIGO IIIC only	No significant association with OS
Skírnisdóttir, I. 2004 [21]	212	Sweden	Monoclonal, clone 113 Novocastra IHC	Percent positivity Membrane localization	No significant association with OS Significantly poorer DFS for FIGO I-II Higher positivity in serous compared to clear cell carcinoma
Stadlmann, S. 2006 [20]	80	Switzerland	Monoclonal, clone 2-18C9 PharmDX (Dako) IHC	Percent positivity Membrane localization Serous tumors only	Significantly associated with EGFR amplification in both primary and recurring tumors
Tanaka, Y. 2011 [42]	102	Japan	Pharm Dx (Dako) IHC	Percent positivity FIGO II, III, IV	No significant association with OS
Wang, K. 2016 [37]	242	China	Polyclonal Santa Cruz IHC	Percent positivity and Intensity	No significant association with OS Significantly poorer OS associated with tumor stroma expression
Wittinger, M. 2011 [25]	144	Austria	Polyclonal Santa Cruz 1:100 IHC	Percent positivity and Intensity	Significantly poorer OS
Xia, W. 2009 [43]	221	USA, Texas	Nuclear stain: Polyclonal Upstate 1:150 Cytoplasmic stain: Clone EGFR.25 Novocastra IHC	Percent positivity and Intensity	Significantly poorer OS with nuclear localization No significant association with OS in cytoplasmic stain
Zhang, M 2015 [27]	161	China	Polyclonal Santa Cruz 1:100 IHC	Percent positivity and Intensity Membrane localization	Significantly poorer OS Significantly higher expression in serous and endometrioid tumors

OS- overall survival, DFS – disease free survival, PFS – progression free survival, HR – hazard ratio. IHC- immunohistochemistry, IF- immunofluorescence

A majority of studies applying morphological criteria have described their tissue as EGFR positive when staining occurs in the cell membrane [9, 18-21, 23, 26, 27, 30-36], while others have included cytoplasmic staining or mixed cytoplasmic/membranous staining [15, 24, 28, 29] or have specifically evaluated staining in the tumor stroma [37]. However, there remain unanswered questions about the biological significance of the predominant staining localization. While membranous positioning of EGFR could allow for higher activation through growth factors and thus more activation of downstream signaling pathways [38], there is also evidence in other tumors that the cytoplasmic localization is associated with an equally malignant phenotype [39, 40].

Despite differing approaches to detecting and scoring EGFR, the majority of studies, similarly to the present report, have concluded that EGFR staining is likely to be of no or only modest utility as a prognostic marker [18, 19, 23, 24, 26, 31, 34, 36, 41, 42]. Few studies showed a significant association of EGFR with poorer overall or disease free survival or with progression in their general patient population, although some significant differences were noted in patient subsets or with multivariate analyses [21, 25, 29, 30, 32, 43, 44] (Table 6). For example in a large Danish study, Nielsen et al. reported significant association of EGFR with poorer overall survival after adjusting for age, FIGO stage, grade, and histotype. However, the HR was modest (1.2), and in a model with more robust molecular markers p53 and HER-2, EGFR offered no additional prognostic effect; the authors concluded that EGFR does not represent an important prognostic factor [44]. In a Swedish study, EGFR staining was significantly associated with progression free survival only in FIGO stage I-II patients [21]. Two studies found EGFR staining to be associated with poorer overall survival in cohorts limited to patients with ovarian serous carcinoma [30, 35]. In a Japanese study, while EGFR staining alone was not prognostic for overall or progression free survival, co-staining of EGFR with another marker, GPR30, was significantly associated with poorer progression free survival [28]. In other studies, significant associations of EGFR with overall survival were seen when using immunofluorescence staining [16], when detecting membranous staining using a novel antibody recognizing the intracellular domain of EGFR [32], or when scoring nuclear stain specifically [29, 43].

The potential use of EGFR as a therapeutic target and tissue staining as a method of patient stratification to select for specific treatments has prompted studies to evaluate possible differences in EGFR protein expression within the various histotypes. Although some studies reported significant differences in staining frequencies by histology, for example in serous tumors compared to clear cell histology [21], serous and endometrioid compared to other histotypes [27], mucinous and serous tumors compared to cystadenomas [9], or in tumors compared to borderline malignancies [28], most found no significant differences in EGFR staining among histotypes [15, 17, 23, 32, 44], as in the present study.

Overall, our results and the review of the literature suggest that the prognostic value of EGFR in ovarian cancer cannot be determined by immunohistochemistry alone. Increased biological understanding of EGFR localization and/or expression levels, as well as improvements in antibodies and image analysis methods, will be necessary to develop specific analysis tools towards improved patient management.

## DISCUSSION

Our study represents one of the largest ovarian cancer patient cohorts assessed by immunohistochemistry for EGFR protein expression and localization. In our previous work with this patient cohort, we found a serine protease inhibitor (SPINK1) to be a prognostic factor for nonserous ovarian tumors; subsequent studies using cell culture models determined that SPINK1-driven ovarian cancer cell proliferation is mediated through EGFR signaling pathways [45]. Given the association between SPINK1 expression and survival for a subset of patients, we assessed potential interrelation between SPINK1 and EGFR staining, but did not find any significant associations (data not shown). Here, we investigated the use of EGFR staining as a single prognostic marker in the same ovarian cancer patient cohort. We found almost 90% of our tumor tissue samples to have some EGFR staining; however, after multiple analyses, we found no significant association with indicators for progression (grade or stage), survival, or histotype. These findings are in general accordance with what has been described in most of the previous studies when looking at overall populations and EGFR staining as a prognostic marker in ovarian cancer, while some studies using smaller patient cohorts or restricted patient subsets have reported statistically significant associations (Table 6).

Subcellular localization of EGFR has been associated with outcome in a number of other tumor types. In pancreatic cancer, shorter overall survival was found for patients with EGFR staining of the tumor cell cytoplasm [39, 46]. In contrast to many other tumors, EGFR localization to the membrane was found to be significantly associated with better patient survival in renal cell carcinoma [47, 48]. In NSCLC, there is some evidence that nuclear EGFR staining may be associated with poor survival [49]. In addition, EGFR membranous expression could be a useful predictive tool for targeted EGFR inhibitor therapy in patients with NSCLC [50]. Differences in associations between tumor types may be due to differential effects of growth factor stimulation with internalized EGFR [39, 51]. The literature on ovarian cancer patients reports cytoplasmic membranous [21], predominant membranous [9], or combined cytoplasmic and membranous stain [28], but we found that dichotomizing the tissue samples based on membranous and cytoplasmic staining did not reveal significant correlation with stage, grade, or patient survival.

While EGFR signaling is involved in promoting ovarian cancer cell proliferation [45], the results of the present study are in agreement with a number of previous analyses showing that EGFR tumor tissue staining by immunohistochemistry may be unpredictive of tumor progression [18, 19, 28, 33, 52]. Alternatives may be to analyze samples via an immunofluorescence staining approach [16], which may provide higher sensitivity and a broader dynamic range relative to immunohistochemistry methods, or by fluorescence in situ hybridization (FISH), which can detect EGFR gene amplification and copy number gain, a measure potentially more closely associated with poor prognosis in ovarian cancer [13, 20, 35].

Efforts using EGFR inhibitors in ovarian cancer patient clinical trials within the general patient population have had only very limited success [8, 38, 53]. One possible explanation is that despite possessing highly elevated levels of EGFR protein, ovarian tumors present only rarely with EGFR mutations, while response to EGFR TKIs in other tumor types such as non-small cell lung cancer (NSCLC) is highly dependent on the presence of mutated EGFR [54, 55]. Mutational screening is also a useful approach to patient stratification in metastatic colorectal carcinoma, where mutations in EGFR pathway mediators *KRAS*, *NRAS*, *BRAF*, and *PIK3CA* are negative predictors of efficacy for anti-EGFR therapeutics [56-58]. However, such mutations are relatively rare in ovarian cancer, and did not predict drug response in the concluded phase III trial of erlotinib in ovarian cancer patients [41]. There may be room for applications of yet untested EGFR modulating drugs or strategies in ovarian cancer patients, but this will likely require a different approach for patient stratification, as current investigation shows that EGFR staining is not consistently associated with tumor response [13].

Other immunohistochemical markers downstream of EGFR signaling pathways such as pAKT, pERK (also known as pMAPK), or pSTAT3 could potentially be more useful as prognostic markers and might also help to stratify ovarian cancer patient populations for treatment with TKIs [59]. These mediators become phosphorylated in the process of activation which can be assessed by tissue staining, but studies are conflicting in terms of the utility of these proteins as prognostic biomarkers. In ovarian cancer patients, high pAKT, high pERK, or their combination have been linked with poor overall survival and progression free survival [41, 42, 60]; however, contrasting studies have failed to find significant associations of pAKT or pERK with survival [19, 23, 61]. High pSTAT3 has also been associated with poorer overall survival in ovarian cancer [62]. While limited studies to date have not been encouraging with regard to the use of pAKT or pERK to predict TKI response in ovarian cancer patients [41], in some other tumor types these markers have shown more promise. In NSCLC for example, high levels of pAKT predicted better response to TKI (gefinitinib) therapy and significantly longer time to progression in one study [63], and in another study, pAKT and pSTAT3 both showed a trend towards association with longer time to progression on gefitinib [64]. While data evaluating the predictive potential of EGFR and related markers in ovarian cancer have thus far not been encouraging, it remains possible that EGFR expression or gene copy number in combination with other markers may yet become useful for stratification of response to treatment.

A strength of the present study is the large patient cohort and the extensively documented patient data that includes clinical history and clinico-pathological details. An additional strength is the application of multiple methods of scoring and analysis enabling relevant comparison with prior studies. Limitations include the relative geographic and ethnic homogeneity of our patient cohort, as some contrasting findings in other studies may reflect population-specific differences.

In conclusion, our results and the current literature indicate that EGFR may not be a robust or generally applicable prognostic immunohistochemical marker for ovarian cancer patients. The success in other cancer types of alternative biomarkers, including activated proteins downstream of EGFR signaling, EGFR mutations and mutations in other pathway genes, may suggest more fruitful directions for identifying potential surrogate markers of EGFR expression, activation, and treatment response in ovarian cancer.

## MATERIALS AND METHODS

### Study population

Tumor biospecimens used for this study were derived from a Mayo Clinic consecutive cohort of 570 patients. Study eligibility included women 20 years or older diagnosed with pathologically confirmed invasive epithelial ovarian, primary peritoneal, or fallopian tube cancer. Patients were enrolled from 1999 to 2009 and were drawn from Mayo Clinic's gynecologic surgery and medical oncology departments. Patients provided written informed consent and protocol procedures and patient contact materials were reviewed and approved by the Institutional Review Board of the Mayo Clinic. All medical records were reviewed and data extracted by experienced research nurses under supervision of gynecologic and medical oncologists. Further details about this cohort have been described previously [65, 66].

### Tissue microarrays and immunohistochemistry

Formalin-fixed, paraffin embedded (FFPE) tumor biospecimens were assembled into five tissue microarrays. Specimen collection and eligibility was coordinated through the Mayo Clinic Ovarian Cancer SPORE and has been previously described [45, 65, 66]. Briefly, tissue cores (0.6mm diameter) were assembled at random placing 350 spots (three cores per patient tumor) with the automated Beecher Instruments ATA-27 arrayer. 5 um sections were cut and mounted on charged slides. Following deparaffinization and rehydration, antigen was retrieved in citrate buffer, endogenous peroxidase was blocked with 3% H_2_O2 and slides were incubated with serum-free protein block (Dako). Slides were then stained for 1 h at room temperature with anti-EGFR [EP38Y] monoclonal antibody (Abcam # ab52894, dilution 1:200) followed by 30 min with secondary anti-rabbit labeled polymer/horse radish peroxidase conjugate (Dako #K4003) finally the color was developed using 3,3′-diaminobenzidine (DAB, EnVision+, Dako).

Stained slides were scanned (ScanScope scanner, Aperio Technologies, Vista, CA), and tissue quality and presence of tumor was determined by CM in consultation with a gynecologic pathologist (AN). Spots with more than 50% tissue damage or fewer than 30 tumor cells were excluded from analysis. Out of 570 patients, 63 were excluded due to missing or damaged tissue in all cores, and 19 were excluded for histological criteria (tumor morphology classified as non-epithelial ovarian, borderline, or unknown). Staining was assessed by scoring tissue cores according to localization (none, cytoplasmic, or membranous; if both cytoplasmic and membranous stain were present the spot was scored as membranous) and a positive pixel count algorithm which gives numeric value corresponding to the % of pixels with moderate or strong staining (Image Scope Software, Aperio Technologies; settings: Hue Value 0.1, Hue width 0.5, Color Saturation Threshold 0.04, lwp (High) 225, lwp(low)=lp(High) 165, lp(low)=Isp(High) 100, Isp(low) 0, Inp(High) -1). The resulting percentages were then dichotomized and defined as low EGFR (<10%) and high EGFR (≥10%). Three cores per patient were stained and the maximum stain value per patient was used for analysis.

### Statistical analysis

Statistical analyses were done using the R statistical software package (version 3.1.1). Associations between EGFR and morphology, stage, and grade were assessed via contingency tables and the Chi-square test. Association of overall survival was assessed via Kaplan Meier curves and Cox proportional hazards models. Models were run both unadjusted and adjusted for stage (early vs. advanced), and debulking status (sub-optimal vs. optimal).

## Abbreviations

EGFR: Epidermal growth factor receptor
SPINK1: serine protease inhibitor kazal type 1

## References

[1] Siegel RL, Miller KD, Jemal A (2016). Cancer statistics, 2016. CA: a cancer journal for clinicians.

[2] Gui T, Shen K (2012). The epidermal growth factor receptor as a therapeutic target in epithelial ovarian cancer. Cancer Epidemiol.

[3] Greenhalgh J, Dwan K, Boland A, Bates V, Vecchio F, Dundar Y, Jain P, Green JA (2016). First-line treatment of advanced epidermal growth factor receptor (EGFR) mutation positive non-squamous non-small cell lung cancer. Cochrane Database Syst Rev.

[4] Tan DSW, Yom SS, Tsao MS, Pass HI, Kelly K, Peled N, Yung RC, Wistuba II, Yatabe Y, Unger M, Mack PC, Wynes MW, Mitsudomi T (2016). The International Association for the Study of Lung Cancer Consensus Statement on Optimizing Management of EGFR Mutation-Positive Non-Small Cell Lung Cancer: Status in 2016. Journal of Thoracic Oncology.

[5] Hynes NE, Lane HA (2005). ERBB receptors and cancer: the complexity of targeted inhibitors. Nat Rev Cancer.

[6] Philip PA, Lutz MP (2015). Targeting Epidermal Growth Factor Receptor-Related Signaling Pathways in Pancreatic Cancer. Pancreas.

[7] Heinemann V, Haas M, Boeck S (2012). Systemic treatment of advanced pancreatic cancer. Cancer Treatment Reviews.

[8] Teplinsky E, Muggia F (2015). EGFR and HER2: is there a role in ovarian cancer?. Translational Cancer Research.

[9] Niikura H, Sasano H, Sato S, Yajima A (1997). Expression of epidermal growth factor-related proteins and epidermal growth factor receptor in common epithelial ovarian tumors. Int J Gynecol Pathol.

[10] Posadas EM, Liel MS, Kwitkowski V, Minasian L, Godwin AK, Hussain MM, Espina V, Wood BJ, Steinberg SM, Kohn EC (2007). A phase II and pharmacodynamic study of gefitinib in patients with refractory or recurrent epithelial ovarian cancer. Cancer.

[11] Gordon AN, Finkler N, Edwards RP, Garcia AA, Crozier M, Irwin DH, Barrett E (2005). Efficacy and safety of erlotinib HCl, an epidermal growth factor receptor (HER1/EGFR) tyrosine kinase inhibitor, in patients with advanced ovarian carcinoma: results from a phase II multicenter study. Int J Gynecol Cancer.

[12] Schilder RJ, Sill MW, Chen X, Darcy KM, Decesare SL, Lewandowski G, Lee RB, Arciero CA, Wu H, Godwin AK (2005). Phase II study of gefitinib in patients with relapsed or persistent ovarian or primary peritoneal carcinoma and evaluation of epidermal growth factor receptor mutations and immunohistochemical expression: a Gynecologic Oncology Group Study. Clinical Cancer Research.

[13] Vergote IB, Jimeno A, Joly F, Katsaros D, Coens C, Despierre E, Marth C, Hall M, Steer CB, Colombo N, Lesoin A, Casado A, Reinthaller A, Green J (2014). Randomized phase III study of erlotinib Versus observation in patients with no evidence of disease progression after first-line platin-based chemotherapy for ovarian carcinoma: a European Organisation for Research and Treatment of Cancer-Gynaecological Cancer Group, and Gynecologic Cancer Intergroup study. J Clin Oncol.

[14] Cho KR, Shih IM (2009). Ovarian Cancer. Annual Review of Pathology.

[15] Lin CK, Chao TK, Yu CP, Yu MH, Jin JS (2009). The expression of six biomarkers in the four most common ovarian cancers: correlation with clinicopathological parameters. Apmis.

[16] Psyrri A, Kassar M, Yu Z, Bamias A, Weinberger PM, Markakis S, Kowalski D, Camp RL, Rimm DL, Dimopoulos MA (2005). Effect of epidermal growth factor receptor expression level on survival in patients with epithelial ovarian cancer. Clinical Cancer Research.

[17] Henzen-Logmans SC, van der Burg ME, Foekens JA, Berns PM, Brussee R, Fieret JH, Klijn JG, Chadha S, Rodenburg CJ (1992). Occurrence of epidermal growth factor receptors in benign and malignant ovarian tumors and normal ovarian tissues: an immunohistochemical study. J Cancer Res Clin Oncol.

[18] Davies S, Holmes A, Lomo L, Steinkamp MP, Kang H, Muller CY, Wilson BS (2014). High incidence of ErbB3, ErbB4, and MET expression in ovarian cancer. Int J Gynecol Pathol.

[19] de Graeff P, Crijns AP, KA Ten Hoor, Klip HG, Hollema H, Oien K, Bartlett JM, Wisman GB, de Bock GH, de Vries EG, de Jong S, van der Zee AG (2008). The ErbB signalling pathway: protein expression and prognostic value in epithelial ovarian cancer. Br J Cancer.

[20] Stadlmann S, Gueth U, Reiser U, Diener PA, Zeimet AG, Wight E, Mirlacher M, Sauter G, Mihatsch MJ, Singer G (2006). Epithelial growth factor receptor status in primary and recurrent ovarian cancer. Mod Pathol.

[21] Skirnisdottir I, Seidal T, Sorbe B (2004). A new prognostic model comprising p53, EGFR, and tumor grade in early stage epithelial ovarian carcinoma and avoiding the problem of inaccurate surgical staging. Int J Gynecol Cancer.

[22] AE Wahner Hendrickson, Hawthorne KM, Goode EL, Kalli KR, Goergen KM, Bakkum-Gamez JN, Cliby WA, Keeney GL, Visscher DW, Tarabishy Y, Oberg AL, Hartmann LC, Maurer MJ (2015). Assessment of published models and prognostic variables in epithelial ovarian cancer at Mayo Clinic. Gynecol Oncol.

[23] Castellvi J, Garcia A, Rojo F, Ruiz-Marcellan C, Gil A, Baselga J, Ramon y Cajal S (2006). Phosphorylated 4E binding protein 1: a hallmark of cell signaling that correlates with survival in ovarian cancer. Cancer.

[24] Lee CH, Huntsman DG, Cheang MC, Parker RL, Brown L, Hoskins P, Miller D, Gilks CB (2005). Assessment of Her-1, Her-2, And Her-3 expression and Her-2 amplification in advanced stage ovarian carcinoma. Int J Gynecol Pathol.

[25] Wittinger M, Vanhara P, El-Gazzar A, Savarese-Brenner B, Pils D, Anees M, Grunt TW, Sibilia M, Holcmann M, Horvat R, Schemper M, Zeillinger R, Schofer C (2011). hVps37A Status affects prognosis and cetuximab sensitivity in ovarian cancer. Clinical Cancer Research.

[26] Elie C, Geay JF, Morcos M, Le Tourneau A, Girre V, Broet P, Marmey B, Chauvenet L, Audouin J, Pujade-Lauraine E, Camilleri-Broet S (2004). Lack of relationship between EGFR-1 immunohistochemical expression and prognosis in a multicentre clinical trial of 93 patients with advanced primary ovarian epithelial cancer (Gineco group). Br J Cancer.

[27] Zhang M, Zhuang G, Sun X, Shen Y, Zhao A, Di W (2015). Risk prediction model for epithelial ovarian cancer using molecular markers and clinical characteristics. Journal of Ovarian Research.

[28] Fujiwara S, Terai Y, Kawaguchi H, Takai M, Yoo S, Tanaka Y, Tanaka T, Tsunetoh S, Sasaki H, Kanemura M, Tanabe A, Yamashita Y, Ohmichi M (2012). GPR30 regulates the EGFR-Akt cascade and predicts lower survival in patients with ovarian cancer. J Ovarian Res.

[29] Alshenawy HA (2010). Immunohistochemical expression of epidermal growth factor receptor, E-cadherin, and matrix metalloproteinase-9 in ovarian epithelial cancer and relation to patient deaths. Annals of Diagnostic Pathology.

[30] Brustmann H (2008). Epidermal growth factor receptor expression in serous ovarian carcinoma: an immunohistochemical study with galectin-3 and cyclin D1 and outcome. Int J Gynecol Pathol.

[31] Demir L, Yigit S, Sadullahoglu C, Akyol M, Cokmert S, Kucukzeybek Y, Alacacioglu A, Cakalagaoglu F, Tarhan MO (2014). Hormone receptor, HER2/NEU and EGFR expression in ovarian carcinoma-is here a prognostic phenotype?. Asian Pac J Cancer Prev.

[32] Noske A, Schwabe M, Weichert W, Darb-Esfahani S, Buckendahl A-C, Sehouli J, Braicu EI, Budczies J, Dietel M, Denkert C (2011). An intracellular targeted antibody detects EGFR as an independent prognostic factor in ovarian carcinomas. BMC Cancer.

[33] Amrani M, Memeo L, Kadiri H, Charhi H, Belabbas MA, Mansukhani MM (2014). Immunohistochemical Analysis of WT1, EGFR, E-cadherin, beta-catenin and p53 in 43 Moroccan Epithelial Ovarian Tumours. Biomedical Engineering Research.

[34] Engelstaedter V, Boda J, Volklein C, Engel J, Jeschke U, Kirchner T, Mayr D (2012). Lack of prognostic relevance of Her-2/neu, topoisomerase IIalpha and EGFR in advanced ovarian carcinoma. Exp Ther Med.

[35] Lassus H, Sihto H, Leminen A, Joensuu H, Isola J, Nupponen NN, Butzow R (2006). Gene amplification, mutation, and protein expression of EGFR and mutations of ERBB2 in serous ovarian carcinoma. J Mol Med.

[36] Raspollini MR, Castiglione F, Garbini F, Villanucci A, Amunni G, Baroni G, Boddi V, Taddei GL (2005). Correlation of epidermal growth factor receptor expression with tumor microdensity vessels and with vascular endothelial growth factor expression in ovarian carcinoma. Int J Surg Pathol.

[37] Wang K, Li D, Sun L (2016). High levels of EGFR expression in tumor stroma are associated with aggressive clinical features in epithelial ovarian cancer. Onco Targets Ther.

[38] Siwak DR, Carey M, Hennessy BT, Nguyen CT, MJ McGahren Murray, Nolden L, Mills GB (2010). Targeting the epidermal growth factor receptor in epithelial ovarian cancer: current knowledge and future challenges. J Oncol.

[39] Einama T, Ueda S, Tsuda H, Ogasawara K, Hatsuse K, Matsubara O, Todo S, Yamamoto J (2012). Membranous and cytoplasmic expression of epidermal growth factor receptor in metastatic pancreatic ductal adenocarcinoma. Exp Ther Med.

[40] Valsecchi ME, McDonald M, Brody JR, Hyslop T, Freydin B, Yeo CJ, Solomides C, Peiper SC, Witkiewicz AK (2012). Epidermal growth factor receptor and insulinlike growth factor 1 receptor expression predict poor survival in pancreatic ductal adenocarcinoma. Cancer.

[41] Despierre E, Vergote I, Anderson R, Coens C, Katsaros D, Hirsch FR, Boeckx B, Varella-Garcia M, Ferrero A, Ray-Coquard I, Berns EM, Casado A, Lambrechts D (2015). Epidermal Growth Factor Receptor (EGFR) Pathway Biomarkers in the Randomized Phase III Trial of Erlotinib Versus Observation in Ovarian Cancer Patients with No Evidence of Disease Progression after First-Line Platinum-Based Chemotherapy. Target Oncol.

[42] Tanaka Y, Terai Y, Tanabe A, Sasaki H, Sekijima T, Fujiwara S, Yamashita Y, Kanemura M, Ueda M, Sugita M, Franklin WA, Ohmichi M (2011). Prognostic effect of epidermal growth factor receptor gene mutations and the aberrant phosphorylation of Akt and ERK in ovarian cancer. Cancer Biol Ther.

[43] Xia W, Wei Y, Du Y, Liu J, Chang B, Yu YL, Huo LF, Miller S, Hung MC (2009). Nuclear expression of epidermal growth factor receptor is a novel prognostic value in patients with ovarian cancer. Mol Carcinog.

[44] Nielsen JS, Jakobsen E, Holund B, Bertelsen K, Jakobsen A (2004). Prognostic significance of p53, Her-2, and EGFR overexpression in borderline and epithelial ovarian cancer. Int J Gynecol Cancer.

[45] Mehner C, Oberg AL, Kalli KR, Nassar A, Hockla A, Pendlebury D, Cichon MA, Goergen KM, Maurer MJ, Goode EL, Keeney GL, Jatoi A, Sahin-Toth M (2015). Serine protease inhibitor Kazal type 1 (SPINK1) drives proliferation and anoikis resistance in a subset of ovarian cancers. Oncotarget.

[46] Ueda S, Ogata S, Tsuda H, Kawarabayashi N, Kimura M, Sugiura Y, Tamai S, Matsubara O, Hatsuse K, Mochizuki H (2004). The correlation between cytoplasmic overexpression of epidermal growth factor receptor and tumor aggressiveness: poor prognosis in patients with pancreatic ductal adenocarcinoma. Pancreas.

[47] Kankaya D, Kiremitci S, Tulunay O, Baltaci S (2016). Prognostic impact of epidermal growth factor receptor on clear cell renal cell carcinoma: Does it change with different expression patterns?. Indian Journal of Pathology and Microbiology.

[48] Kallio JP, Hirvikoski P, Helin H, Kellokumpu-Lehtinen P, Luukkaala T, Tammela TL, Martikainen PM (2003). Membranous location of EGFR immunostaining is associated with good prognosis in renal cell carcinoma. Br J Cancer.

[49] Traynor AM, Weigel TL, Oettel KR, Yang DT, Zhang C, Kim K, Salgia R, Iida M, Brand TM, Hoang T, Campbell TC, Hernan HR, Wheeler DL (2013). Nuclear EGFR protein expression predicts poor survival in early stage non-small cell lung cancer. Lung Cancer.

[50] Pirker R, Pereira JR, von Pawel J, Krzakowski M, Ramlau R, Park K, de Marinis F, Eberhardt WEE, Paz-Ares L, Störkel S, Schumacher K-M, von Heydebreck A, Celik I (2012). EGFR expression as a predictor of survival for first-line chemotherapy plus cetuximab in patients with advanced non-small-cell lung cancer: analysis of data from the phase 3 FLEX study. The Lancet Oncology.

[51] Willmarth NE, Baillo A, Dziubinski ML, Wilson K, Riese DJ, Ethier SP (2009). Altered EGFR Localization and Degradation in Human Breast Cancer Cells with an Amphiregulin/EGFR Autocrine Loop. Cell Signal.

[52] de Graeff P, Crijns AP, de Jong S, Boezen M, Post WJ, de Vries EG, van der Zee AG, de Bock GH (2009). Modest effect of p53, EGFR and HER-2/neu on prognosis in epithelial ovarian cancer: a meta-analysis. Br J Cancer.

[53] Murphy M, Stordal B (2011). Erlotinib or gefitinib for the treatment of relapsed platinum pretreated non-small cell lung cancer and ovarian cancer: a systematic review. Drug Resist Updat.

[54] Vargas AJ, Harris CC (2016). Biomarker development in the precision medicine era: lung cancer as a case study. Nat Rev Cancer.

[55] Lee CK, Brown C, Gralla RJ, Hirsh V, Thongprasert S, Tsai CM, Tan EH, Ho JC, T Chu da, Zaatar A, JA Osorio Sanchez, Vu VV, Au JS, Inoue A, Lee SM, Gebski V (2013). Impact of EGFR inhibitor in non-small cell lung cancer on progression-free and overall survival: a meta-analysis. J Natl Cancer Inst.

[56] Nicolantonio FD, Martini M, Molinari F, Sartore-Bianchi A, Arena S, Saletti P, Dosso SD, Mazzucchelli L, Frattini M, Siena S, Bardelli A (2008). Wild-Type BRAF Is Required for Response to Panitumumab or Cetuximab in Metastatic Colorectal Cancer. Journal of Clinical Oncology.

[57] De Roock W, Claes B, Bernasconi D, De Schutter J, Biesmans B, Fountzilas G, Kalogeras KT, Kotoula V, Papamichael D, Laurent-Puig P (2010). Effects of KRAS, BRAF, NRAS, and PIK3CA mutations on the efficacy of cetuximab plus chemotherapy in chemotherapy-refractory metastatic colorectal cancer: a retrospective consortium analysis. The lancet oncology.

[58] Cutsem EV, Köhne C-H, Láng I, Folprecht G, Nowacki MP, Cascinu S, Shchepotin I, Maurel J, Cunningham D, Tejpar S, Schlichting M, Zubel A, Celik I (2011). Cetuximab Plus Irinotecan, Fluorouracil, and Leucovorin As First-Line Treatment for Metastatic Colorectal Cancer: Updated Analysis of Overall Survival According to Tumor KRAS and BRAF Mutation Status. Journal of Clinical Oncology.

[59] Seshacharyulu P, Ponnusamy MP, Haridas D, Jain M, Ganti A, Batra SK (2012). Targeting the EGFR signaling pathway in cancer therapy. Expert Opin Ther Targets.

[60] Wang Y, Kristensen GB, Helland A, Nesland JM, Borresen-Dale AL, Holm R (2005). Protein expression and prognostic value of genes in the erb-b signaling pathway in advanced ovarian carcinomas. Am J Clin Pathol.

[61] Woenckhaus J, Steger K, Sturm K, Munstedt K, Franke FE, Fenic I (2007). Prognostic value of PIK3CA and phosphorylated AKT expression in ovarian cancer. Virchows Arch.

[62] Min H, Wei-hong Z (2009). Constitutive activation of signal transducer and activator of transcription 3 in epithelial ovarian carcinoma. J Obstet Gynaecol Res.

[63] Cappuzzo F, Magrini E, Ceresoli GL, Bartolini S, Rossi E, Ludovini V, Gregorc V, Ligorio C, Cancellieri A, Damiani S, Spreafico A, Paties CT, Lombardo L (2004). Akt phosphorylation and gefitinib efficacy in patients with advanced non-small-cell lung cancer. J Natl Cancer Inst.

[64] Emery IF, Battelli C, Auclair PL, Carrier K, Hayes DM (2009). Response to gefitinib and erlotinib in Non-small cell lung cancer: a restrospective study. BMC Cancer.

[65] Goode EL, Chenevix-Trench G, Hartmann LC, Fridley BL, Kalli KR, Vierkant RA, Larson MC, White KL, Keeney GL, Oberg TN, Cunningham JM, Beesley J, Johnatty SE (2011). Assessment of hepatocyte growth factor in ovarian cancer mortality. Cancer Epidemiol Biomarkers Prev.

[66] Goode EL, Maurer MJ, Sellers TA, Phelan CM, Kalli KR, Fridley BL, Vierkant RA, Armasu SM, White KL, Keeney GL, Cliby WA, Rider DN, Kelemen LE (2010). Inherited determinants of ovarian cancer survival. Clinical Cancer Research.

